# Molecular Principles of Insect Chemoreception

**DOI:** 10.32607/actanaturae.11038

**Published:** 2020

**Authors:** E. L. Sokolinskaya, D. V. Kolesov, K. A. Lukyanov, A. M. Bogdanov

**Affiliations:** Shemyakin-Ovchinnikov Institute of Bioorganic Chemistry, Moscow, 117997 Russia

**Keywords:** chemoreceptor, cation channel, action potential, ionotropic receptor, metabotropic receptor, odorant, olfaction, gustatory receptor, insects

## Abstract

Chemoreception, an ability to perceive specific chemical stimuli, is one of the most evolutionarily ancient forms of interaction between living organisms and their environment. Chemoreception systems are found in organisms belonging to all biological kingdoms. In higher multicellular animals, chemoreception (along with photo- and mechanoreception) underlies the functioning of five traditional senses. Insects have developed a peculiar and one of the most sophisticated chemoreception systems, which exploits at least three receptor superfamilies providing perception of smell and taste, as well as chemical communication in these animals. The enormous diversity of physiologically relevant compounds in the environment has given rise to a wide-ranging repertoire of chemoreceptors of various specificities. Thus, in insects, they are represented by several structurally and functionally distinct protein classes and are encoded by hundreds of genes. In the current review, we briefly characterize the insect chemoreception system by describing the main groups of receptors that constitute it and putting emphasis on the peculiar architecture and mechanisms of functioning possessed by these molecules.

## INTRODUCTION

Living creatures get information about their environment via the senses: vision, hearing, smell, and taste. Perception of environmental factors in each sensory system is mediated by a small region of tissue that is sensitive to a specific physical stimulus (electromagnetic radiation in the case of vision, mechanical vibrations of air in the case of hearing, and chemicals in the case of smell and taste). In multicellular organisms, such specialized tissue structures are called *receptors*. Receptor cells convert captured light, sound, or chemicals into a nerve impulse that is transmitted to the brain for processing of the received information. The conversion of a physical stimulus to a nerve impulse is known as signal transduction. During this process, receptor cells (neurons or other specialized cells) perceive a signal using the special receptor molecules. This changes the activity of ion channels in the neuron plasma membrane and, therefore, causes a shift in the cell membrane voltage (depolarization or hyperpolarization). Depolarization triggers the action potential and then promotes the transmission of a nerve impulse in the nervous system.

Receptor molecules can either directly activate ion channels (in this case, the receptor is called ionotropic) or run a membrane signaling cascade leading to the activation of ion channels through specialized G proteins (in this case, the receptor is called metabotropic). The ionotropic signal transduction pathway reveals its advantages for high-intensity stimuli, since it provides the fastest “start” of a neuronal excitation. On the other hand, metabotropic transduction is indispensable in the case of weak stimuli, whose perception requires signal amplification. The sensory organs of multicellular animals use both mechanisms, sometimes combining them sophisticatedly.

## COMMON PRINCIPLES OF ANIMAL CHEMORECEPTION

**Fig. 1:**
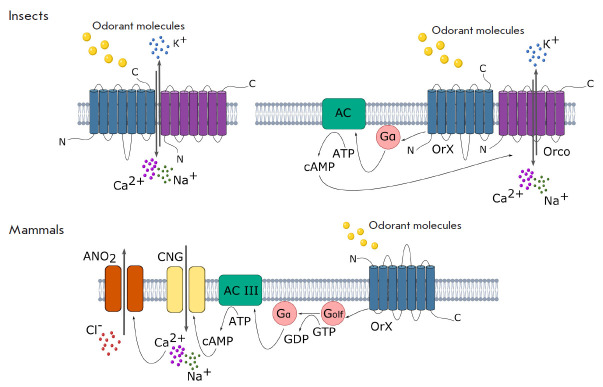
Molecular mechanisms of signal transduction by the olfactory receptors of insects and mammals. The main (ionotropic) and additional (metabotropic) ways of functioning of the insect olfactory receptors are shown in the upper part of the scheme. The mammalian olfactory receptor and the membrane cascade ensuring the transduction of its signal are shown in the lower part of the scheme [2–4]. ACIII – type III adenylate cyclase; ATP – adenosine triphosphate; cAMP – cyclic adenosine monophosphate; ANO_2_ – anoctamin-2 channel; CNG – a cyclic nucleotide-binding channel; Gα – α-subunit of the olfactory G protein; OrX – odor-specific receptor olfactory protein Or; Orco – constant co-receptor olfactory protein; Na^+^, K+, Ca^2+^, Cl^-^ – sodium, potassium, calcium cations and chloride anion, respectively

Chemoreception is an important element in the perception and analysis of environmental information. Chemical stimulation provides recognition of taste and food quality, alerts animals to the presence of potential predators or other dangers, and directs social interactions. Smells, tastes, and other chemical stimuli are recognized and decoded by a diverse set of chemosensory systems in various animals. Chemosensory transduction is a process in which chemical stimuli – smells, tastes, nutrients, irritants, and even gases – are recognized and cause changes in the cell membrane properties or release of neurotransmitters and hormones [1]. Typically, transduction processes occur in sensitive neurons, which often form specialized subcellular compartments (cilia or microvilli) optimized for transduction. In most cases, chemosensory transduction is a multi-stage pathway in which the biochemical signal on the membrane is converted into an electrical signal, the action potential. Chemical signals (or chemical stimuli) are represented by molecules originating from various sources, such as soil, plants, or animals. These compounds can be volatile or in dissolved state. In the first case, the chemical signal is perceived by olfactory receptors; in the second one, by taste (gustatory) receptors. Among the complex chemosensory systems of higher multicellular animals, the olfactory and gustatory analyzers of insects and mammals have been the best studied at the molecular level. Interestingly, when responding to chemical stimulation, mammals rely mainly on metabotropic receptors, while insects rely on ionotropic ones [2] (*Fig. 1*).

## INSECT CHEMORECEPTORS

**Olfactory receptors**

**Fig. 2:**
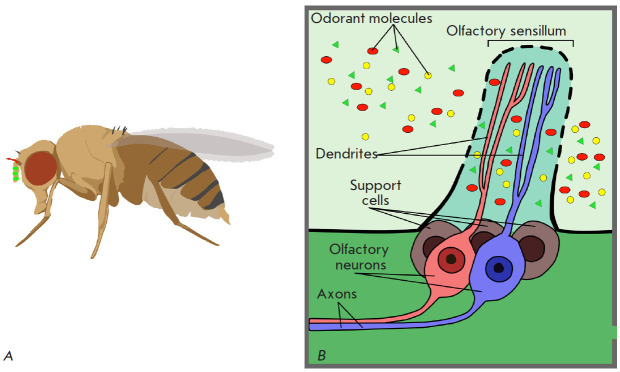
The pathway of the odorant molecules entering the olfactory receptor from the environment. Molecules reach the antennae of the fly equipped with numerous sensitive hairs: sensilla (*A*). The surface of each sensillum has pores that allow odorant molecules to penetrate inside the sensillum, where the dendrites of the olfactory sensitive neurons (OSNs) are located (*B*). The olfactory receptors specifically binding odor molecules are exhibited on dendritic membranes. Adapted from [1]

In insects, olfactory sensory neurons (OSNs), which express olfactory receptors in their dendrites, are responsible for odor perception. OSNs are localized in the forehead appendages, antennae, and maxillary palps (*Fig. 2*).

**Fig. 3:**
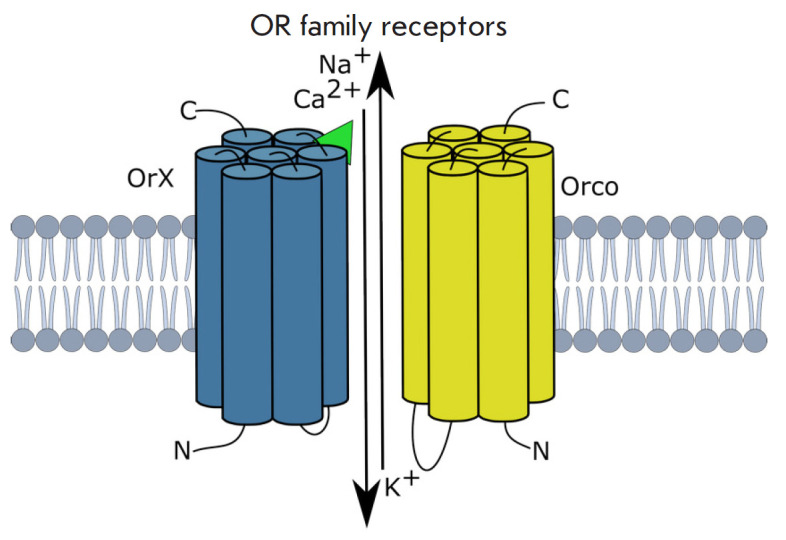
Insect odorant receptors (ORs). ORs – the heterodimers consisting of Orco co-receptor and the odor-specific Or protein: OrX in the case of food odor recognition (also sensitive to odors of oviposition places, predators, toxic substances, etc.) and OrY in the case of pheromone recognition [3]. The green triangle depicts the ligand (an odorant molecule)

The sense of smell in insects is provided by three types of receptors; members of different families of transmembrane proteins. The first type is represented by the odorant (or olfactory) receptors (ORs) that recognize food odors and pheromones. ORs function as heterodimers consisting of a variable odor-specific Or receptor protein and a constant co-receptor Orco protein [5, 6] (*Fig. 3*).

**Fig. 4:**
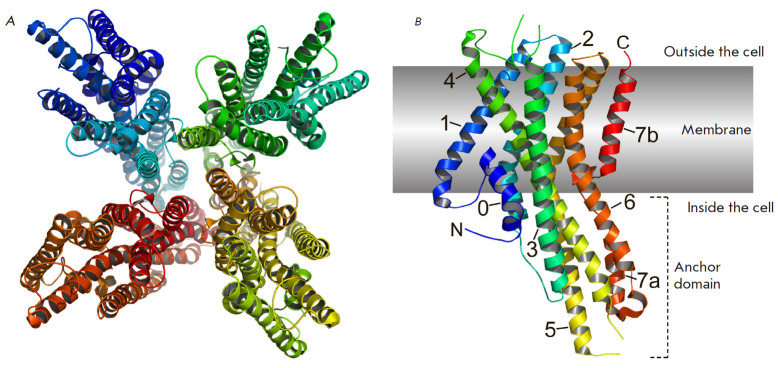
The structure Orco from *Apocrypta bakeri*. (*A*) Structure of the homotetramer, view from the cytoplasmic side. (*B*) Monomer structure. The numbers indicate alpha helices. Structures are represented using the PyMol program based on PBD ID 6C70

Similarly to a typical G protein-conjugated receptor (GPCR), ORs consist of seven associated α-helices; however, they differ from GPCR in terms of helice orientation with respect to the plasma membrane. Thus, insect ORs have a cytoplasmic N-terminus and an extracellular C-terminus [7, 8]. Although the OrX/Y-Orco heterodimer serves as an elementary functional unit of the insect olfactory system, the receptors in the membrane of olfactory neurons most likely function within large supramolecular ensembles, whose composition and topology remain poorly understood. Recent studies have shed light on the molecular organization of such complexes. Thus, the cryo-electron microscopy structure of the Orco subunit from *Apocrypta bakeri* was resolved [9]. Orco forms tetramers having a “pinwheel” shape when viewed from above, perpendicular to the membrane plane (*Fig. 4A*). The tetramer is approximately 100 Å in diameter and 80 Å in the axial direction. The central pore is formed by four subunits. Each subunit has seven helical segments that penetrate the membrane at an angle of ~30°; at the same time, the C-terminus of each subunit is oriented outward of the cell, while the N-terminus has an inward orientation (*Fig. 4B*). In addition to the seven main helices (helices 1–7), there is an extra N-terminal helix (helix 0), which is placed under loop 4 along the outer perimeter of the channel during complex assembly. Helix 7 is closest to the central axis and consists of two parts: the cytoplasmic segment (7a) and the transmembrane segment (7b), which are separated by a β-hairpin consisting of 15 amino acid residues. Helix 7b forms the central pore, and helix 7a forms the core of the anchor domain. Helices 4, 5, and 6 extend far beyond the cell membrane (40 Å into the cytosol), where they surround helix 7a, completing the formation of the anchor domain. The transmembrane domain of each subunit is stabilized by the charged and polar amino acids of helices 2, 4, 5, and 6, thus forming a dense network of hydrophilic interactions within the intracellular leaflet of the membrane. Within the extracellular leaflet, helices 1–6 split to form a cleft 10 Å deep and ~ 20 Å. It is assumed that such a pocket could serve as a binding site for low-molecular-weight ligands. Mutations altering the specificity of ORs for odorants are also mapped within this pocket, indicating that there potentially exists a common structural locus for ligand binding in Orco and OR. In the Orco structure, the ordered region of the extracellular loops 3–4 restricts access to the pocket, which may interfere with odorant binding, thereby preserving odor specificity in the Orco–OR complexes.

The architecture of Or–Orco receptor complexes has not yet been established. The subject of discussion remains the topology of the receptor heterodimer itself. It is assumed that Or proteins can form heterodimers with the Orco co-receptor in a way similar to the seven-helix channelrhodopsin, where the ion channel pore is formed by opposing TM3 and TM4 helices [10, 11]. Another possible assembly option is a tetramer consisting of two dimers [12]. The relative cation permeability varies for different OrX [13, 14]. A mutation analysis of the olfactory silkworm (*Bombyx mori*) receptors showed that the OR channel pore is formed by both types of proteins, OrX and Orco [15]. Expression of Orco proteins alone (in the absence of OrX) also leads to the formation of functional channels that do not bind odorant molecules but can be activated by cyclic nucleotides [16] or synthetic agonists [17, 18, 19].

The molecular mechanism of OR activation has not been investigated in details. In some studies, the exclusively ionotropic mechanism was found [13]; in other studies, the metabotropic signaling mechanism based on the DAG/IP3 pathway was clearly shown [20, 21]. Finally, both signaling pathways were detected in heterologously expressed *Drosophila *OR proteins [16].

**Fig. 5:**
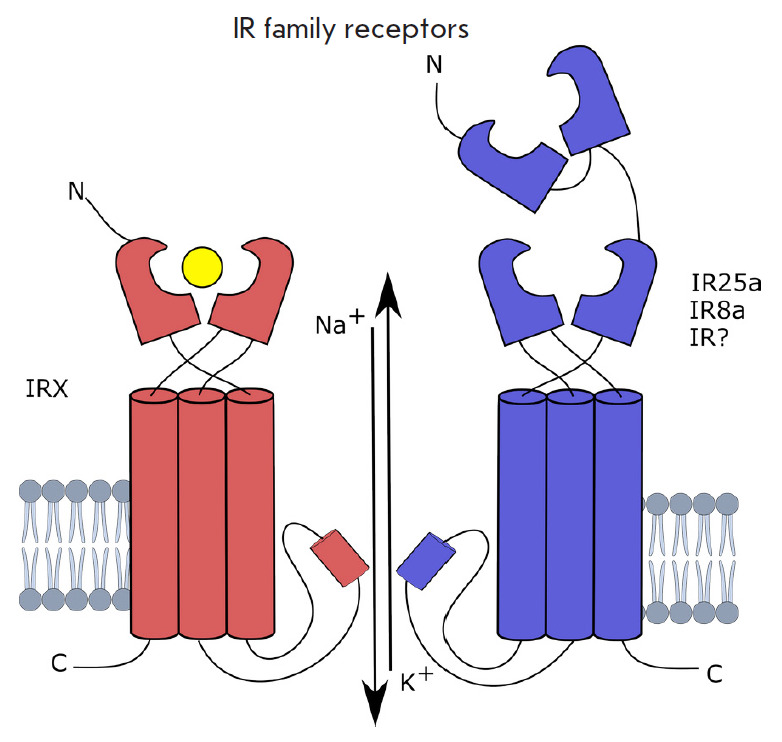
Ionotropic receptors (IRs) are heterotetramers consisting of the IRcoY co-receptor protein and IRX receptor protein [3]

The second type of olfactory insect receptors is represented by the so-called ionotropic receptors (IRs) homologous to ionotropic glutamate receptors (involved in the formation of synaptic contacts in the nervous system of vertebrates and invertebrates) [22]. IRs are sensitive to acids, amines, and aldehydes. Receptors function as heterotetramers consisting of an odor-specific receptor protein IRX and a constant co-receptor protein IRcoY [23] (*Fig. 5*). However, IR receptors acting as heterodimers have also been described. Thus, the IR8a + IR75a heterodimer responds to acetic acid [23, 24, 25]. The IR8a + IR84a pair, whose specificity was characterized in *Xenopus laevis *oocytes, is activated by phenylacetaldehyde [24]. Olfactory sensilla expressing IR8a + IR64a recognize acids and free protons [24, 26]. Artificial stimulation of IR64a-positive neurons causes avoidance behavior, which corresponds to the role of these neurons in acidic stimuli detection. IR8a was shown to be associated with IR64a, thereby contributing to the stability of IR64a [23]. Together, these results indicate that IR8a functions as a co-receptor in IR64a-positive neurons [27].

IRcoY also carries a ligand-binding domain; however, it is suggested that its main role is to traffic the complex onto the cell membrane but not to bind the ligand [23].

IRs can form tetramers consisting of two IRcoY:IRX dimers, or of the IRcoY monomer plus three different IRX subunits. In fruit fly, IR co-receptors are represented by IR8a and IR25a [11]. Both IRcoY and IRX consist of three transmembrane helices separated by an extracellular region containing a ligand-binding domain (LBD). IRcoY also has a massive N-terminal domain (ATD). IRs are non-selective cation channels and, upon activation, conduct Na^+^ and K+ ions, and some of them also Ca^2+^ cations [23].

IRs and ORs recognize odors with a complementary specificity: their ligands do not overlap. Drosophila OR-expressing olfactory neurons have been shown to better adapt to background odors compared to IR-expressing neurons. This feature allows insects to track odor changes over a wide range of concentrations and detect other odors even if a certain background exists. Meanwhile, despite their inability to adapt, IRs more accurately determine the absolute concentration of odorant that allows fruit fly to efficiently track food location, sexual partners, or predators [28, 29]. Most of the IR-family receptors are specifically activated by amines and acids. The IR76b receptor is specific to low NaCl concentrations [30].

**Fig. 6:**
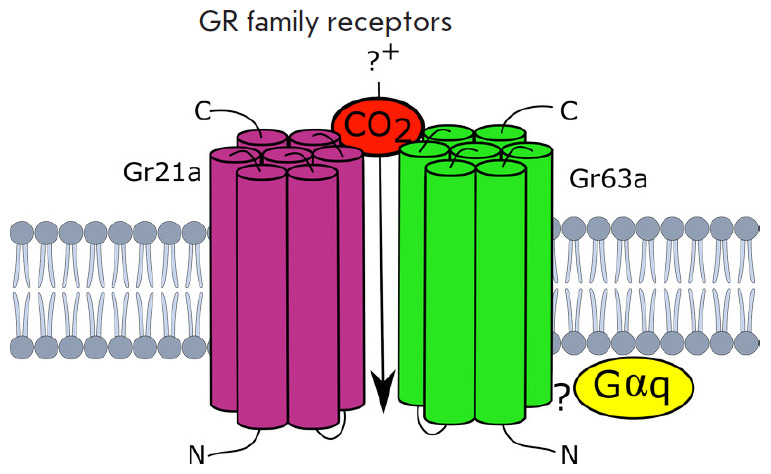
Gustatory receptors (GRs) sensitive to carbon dioxide: heterodimers consisting of the Gr1/Gr2 and Gr3 subunits. GRs have structural and topological motifs that are similar to those in ORs [3]

The third type of olfactory receptors is represented by specialized gustatory receptors (GRs) sensitive to carbon dioxide [31]. Like ORs, GRs belong to the family of seven-transmembrane domain receptors (7TM receptors), with the orientation of transmembrane domains opposite to that of GPCR proteins. Three *Gr *genes encoding receptors sensitive to carbon dioxide were found [32]. Receptors also form heterodimers consisting of Gr1/2 and Gr3 subunits (*Fig. 6*), which are represented in *Drosophila *by the Gr21a and Gr63a proteins, respectively [31, 33].

In *Drosophila*, Gr21a and Gr63a form a complex with Gαq proteins activating phospholipase C, which, in turn, activates the TRP-family ion channel through phosphoinositide hydrolysis [34, 35, 36]. Acidic odors and high carbon dioxide concentrations (> 5%) are recognized by the IR family receptor, namely IR64a [26].

**Gustatory receptors**

**Fig. 7:**
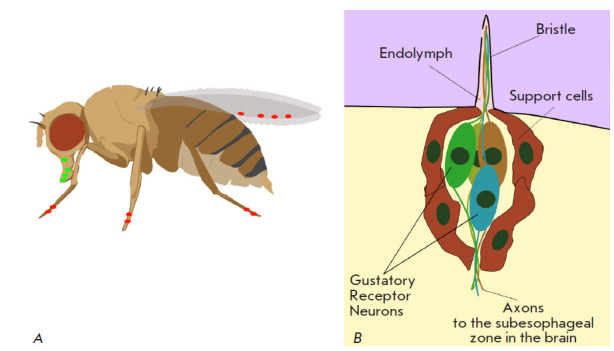
Taste organs of *D. melanogaster*. (A) The main localization points of taste sensilla on the *Drosophila *body (shown with colored dots). (B) Scheme of the cellular organization of taste sensillum. Adapted from [1]

Insects are generally characterized by a complex taste sensory system. The main taste organs, taste sensilla, are located mostly on the legs and wings [37] (*Fig. 7A*). Receptor cells are sensitive neurons, most of which are associated with taste sensilla (*Fig. 7B*). Each sensillum contains several gustatory receptor neurons (GRNs), with gustatory receptors (GRs) expressed in their dendrites.

**Fig. 8:**
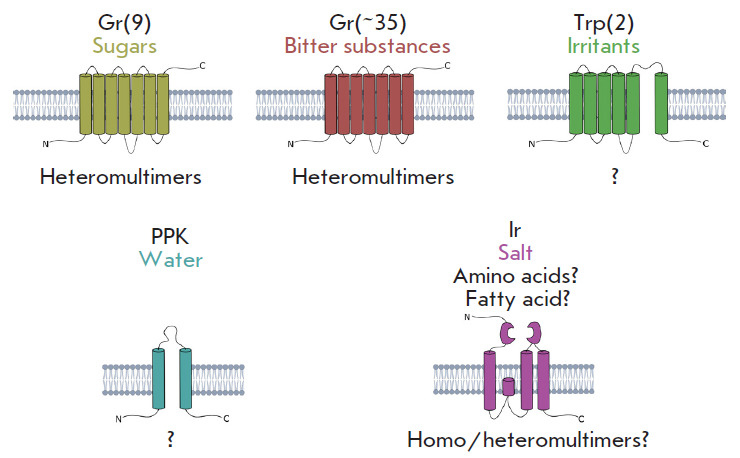
Structures of different gustatory receptors in adult *D. melanogaster*. At least four types of receptors have been detected in taste neurons. Over 40 of the 68 *Gr *genes encode receptors for bitter and sweet taste. Two TRP genes were shown to encode taste-sensitive receptors (aristocholic acid and allyl isothiocyanate). At least one molecule (PPK28 channel) is used to determine the taste of water. The role of IR family receptors in taste perception is poorly understood, but expression in gustatory neurons has been shown for 15 genes. At least one example sensitive to sodium chloride taste (IR76b) has been reported. Adapted from [1]

The discovery of the GR gene family in 2000 [38, 39, 40] was a breakthrough in the study of insect taste behavior and the physiology of their taste perception. The *Drosophila *genome contains 68 *Gr *genes [41], some of which are highly conserved among arthropods [42]. The *Gr *genes can be divided into two large groups. The first group includes most *Gr *genes (about 35), which are expressed in neurons that recognize bitter and salty tastes [43]. The second group consists of eight genes expressed exclusively in neurons sensitive to the sweet taste [44] (*Fig. 8*).

**Gr receptors of sweet taste**

Recognition of the sweet taste of sugars is the best studied form of taste perception in *Drosophila*. Unlike mammals, where the only heterodimeric G protein-coupled receptor complex recognizes all sugars and even sweet-tasting proteins [45, 46], *Drosophila *was found to have eight Gr receptors involved in determining the sweet taste and encoded by the *Gr5a, Gr61a *and a cluster of six *Gr64a-f *genes [44]. All the genes encoding Gr receptors sensitive to the sweet taste are expressed in the paws, with a single exception for *Gr64a*, which is expressed in the palps [44]. Functional sweet taste receptors are heterodimers [47]. However, receptors that can function as homomultimers or monomers (Gr43a-like) are also known [48]. Below one can find a brief description of *Gr* genes encoding sweet taste receptors (*Table 1*) [1]. The data on specificity were obtained based on knockouts of the corresponding genes.

**Table 1:** Brief description of the Gr genes encoding sweet taste receptors

Gene	Ligand	Partner
Gr5a	Trehalose	Gr64f
Gr61a	Glucose	?
Gr64a	Maltose Fructose	Gr64e
Gr64b	Glycerol	Gr64e
Gr64c	Sucrose Maltose Arabinose	?
Gr64e	Glycerol	Gr64a/Gr64b
Gr64f	Glucose Sucrose Fructose Maltose Trehalose Melezitose	Gr5a
Gr43a	Fructose Sucrose	None

**Bitter taste receptors**

Similarly to mammals, *Drosophila *and other insects have systems that are well-tuned to detect potentially dangerous substances, usually bitter and lacking nutritional value. Insects meet a wide range of bitter sub stances from various sources. For example, many plants produce bitter substances as secondary metabolites that they use to protect against herbivorous insects [1]. For *Drosophila* and other insects consuming fruits, microorganisms inhabiting rotting fruits are a source of dangerous bitter substances. Bitter substances are represented by a wide range of components with diverse structures, such as alkaloids, terpenoids, and phenols. Therefore, most of the gustatory receptors (about 35) are sensitive to bitter substances [49]. However, only four bitter taste Gr receptors were functionally characterized. These receptors are presented in the table below (*Table 2*).

**Table 2:** Gr receptors of bitter taste

Gene	Ligand	Partner
Gr8a	L-canavanine	?
Gr66a	Caffeine Diethyltoluamide Papaverine Strychnine Lobeline	?
Gr93a	Caffeine	?
DmRX	L-canavanine	None

Note: L-Canavanin is a non-proteinogenic amino acid found in some leguminous plants; an insecticide. Diethyltoluamide is an artificially synthesized organic compound with a repellent and insecticidal effect.

It is assumed that bitter taste Gr receptors also consist of several subunits, and that Gr33a and Gr66a are the core subunits of such multimeric complexes [49, 50].

**Gustatory signal transduction**

The signaling pathways of gustatory receptors are poorly understood. There are two reasons for the lack of knowledge in this research field. First, the gustatory neurons in *Drosophila *are not susceptible to electrophysiological studies using the patch-clamp method, which makes it impossible to study the neurophysiological processes underlying receptor activation. Second, most of the attempts to express gustatory receptors in a heterologous system have failed. The only exception is representatives of the so-called Gr43a-like clade, a family of receptors classified as taste receptors for fructose by phylogenetic analysis [51].

Available data suggest that insect receptors belonging to the Gr43a-like clade (conserved in many holometabolous insects) are ionotropic homosubunit chemoreceptors. Orthologs of the *DmGr43a *fruit fly gene are represented by *BmGr9 *in *B. mori*, *HarmGr4 *in *H. armigera*, and *AmGr3 *in *A. mellifera*. Recently, *DmGr43a *paralogs have been discovered in *T. castaneum *(*TcGr20*) and *B. mori *(*BmGr10*). In 2011, Japanese scientists succeeded in an heterologous expression of the *BmGr9 *gene from silkworm (*B. mori*) and its ortholog *DmGr43a *from *Drosophila *[52]. The *BmGr9 *gene was expressed in human embryonic kidney cells (HEK293T line) and in *Xenopus *oocytes; *DmGr43a*, in COS-7 cell line (fibroblast-like cells from monkey kidneys). Using the patch-clamp method, the authors showed that D-fructose is the ligand for the BmGr9 and DmGr43a receptors. Recording of the fluorescence dynamics of calcium indicator after D-fructose application confirmed the electrophysiological results. It was shown that the BmGr9 receptor functions as a ligand-gated cation channel: inhibition of G protein-coupled signaling with the U73122 agent (phospholipase C inhibitor) did not prevent the entry of Ca^2+^ ions upon application of fructose onto cells expressing BmGr9. Moreover, stimulation with a cyclic nucleotide analog and adenylate cyclase activator (compounds essential for G protein-coupled signaling) failed to produce a calcium response in BmGr-9–expressing HEK293T cells.

It was later shown that the BmGr10 receptor sensitive to *myo- *and *epi*-inositol (whose gene is a paralogue of BmGr9) is also a ligand-dependent cation channel [53]. The presence of an inward calcium current upon inhibition of G protein cascades with U73122 proved the ionotropic nature of the receptor.

Insect Gr receptors continue to be actively studied. Thus, it has recently been shown that the TcGr20 receptor in *Tribolium castaneum *is sensitive to sorbitol and mannitol [54]. *Tribolium castaneum *(red flour beetle), a common pest of dry goods, has 207 Gr genes. Apparently, such a wide repertoire of gustatory receptors is necessary for universal consumer species (which consume various types of products and are not tied to a specific type of food) (*Table 3*) [54].

**Table 3:** Sweet taste Gr receptors with a reported signal transduction mechanism

Receptor	Ligand	Natural source of receptors	Description of receptors in the literature
BmGr9	D-fructose	Bombyx mori (domestic silkworm)	[52]
BmGr10	myo-inositol epi-inositol	Bombyx mori (domestic silkworm)	[53]
TcGr20	mannitol sorbitol	Tribolium castaneum (red flour beetle)	[54]

Thus, the members of the Gr43a-clade are insect gustatory receptors with the best-studied functioning mechanism. Moreover, the possibility of heterologous expression of their genes and such chemoreceptor properties as ionotropy and homomery provide ample opportunities to study them in detail in various model systems *in cellulo* and *in vitro* and even for the development of electrophysiological instruments based on them.

**The expression features of insect chemoreceptors**

Insects and vertebrates differ not only in the structure of chemoreceptors, but also in their gene expression strategies. Thus, in each of the approximately 10 million vertebral olfactory neurons, strictly one receptor gene is expressed. The implementation of the “one receptor – one neuron” rule is ensured by regulation at the transcriptional level. It is assumed that after the functional type of the receptor expressed in a particular cell is selected, transcription of the remaining receptor genes is suppressed by the feedback principle [55]. The mechanism used by a neuron to “choose” its olfactory receptor and arrest the expression of the receptors of all the other specificities is still poorly understood [56]. Most likely, following the “one receptor – one neuron” rule is important for accurate “decoding” of olfactory signals, which implies that the given population of olfactory neurons responds to a limited number of odorants, and that the olfactory center uniquely identifies the origin of incoming signals [56].

In *Drosophila*, most of the ~2,600 available olfactory neurons express two olfactory receptor genes: one is cell type-specific (odor-specific Or subunit), and the second one is the *Or83b *(Orco co-receptor). Dimerization of Or83b with a specific receptor provides trafficking of the functional complex toward the olfactory sensilla [6]. At the first glance, this principle seems synonymous with the vertebrate “one receptor – one neuron” rule described above; however, *Drosophila *has more flexible expression conditions. For example, in 6 out of 8 classes of olfactory antenna neurons, two genes of the odor-specific Or subunit are expressed in addition to Or83b [57]. Moreover, all the neurons of a particular sensillum always express the same Or receptor, although *Drosophila *has not been found to suppress the expression of other genes through the feedback principle characteristic of vertebrates [58].

In the *Drosophila *genome, the OR protein family is encoded by 60 genes and several pseudogenes. It consists of 62 receptor proteins (*Or46a *and *Or69a *encode two proteins each via alternative splicing [41]). Some OR genes are grouped into clusters of two or three genes (probably because they appeared as a result of duplication), but most genes are widely distributed across the genome [41]. The expression analysis of OR genes showed that 45 members of the family are expressed in the antennae and maxillary palps of adult animals, while 25 genes function only in the larva olfactory system [57].

Interestingly, OR family receptors were found only in flying insects. Some authors suggest that the dual transduction system characteristic of OR is an adaptation to smell source recognition during flight [59, 60].

The IR receptor family is extremely divergent and demonstrates a shared amino acid sequence identity of 10–70%. Like the OR genes, IR genes are scattered throughout the *Drosophila *genome, mainly as individual genes, but some also form clusters [22]. Genomic analysis of *Drosophila *revealed 66 genes belonging to the IR family, including 9 presumable pseudogenes [22]. Notably, 16 representatives of the family are expressed in olfactory antenna neurons (IR receptors sensitive to organic acids and amines), and 44 in the taste organs (32 at the larval stage, 27 in the adult insect) – labellum, legs, pharynx, and the anterior wing margin [61].

The genome of *Drosophila *also contains 60 genes of the GR family, which encode 68 proteins including those produced via alternative splicing [61]. GR proteins are extremely divergent in amino acid sequences (only 8% identity). The GR family members are expressed in the taste organs of adult animals (the labellum, legs, and pharynx), in the gustatory organs of larvae, as well as in various other tissues of adult animals, including antenna, maxillary palps, enteroendocrine cells of the gut, multidendritic cells of the abdominal body wall, in neurons innervating reproductive organs, and even in the brain [61].

Expression of chemoreceptors is affected by the physiological state of the insect’s organism, which, in turn, depends on environmental factors. Thus, a study of the mRNA levels of the 21 IR, 12 GR, and 43 OR receptors in the antennae of *Bactrocera dorsalis *oriental fruit fly revealed that expression significantly depends on the nutritional and sexual behavior of insects and even on the time of day [62]. Interestingly, the direction of regulation and its quantitative characteristics appears to be completely different for receptors of different types and individuals of different sexes. These data presumably illustrate the dynamic adaptation of insect physiology to changes in external conditions, providing a certain degree of flexibility in the implementation of behavioral programs.

An analysis of the transcriptomes of eusocial insects, and *Reticulitermes speratus* termites in particular, revealed a differential expression of the *Or*, *Ir*, and *Gr* genes associated with sex, age, and specialization (caste affiliation) of the studied individuals [63]. It is likely that similar expression features may be characteristic of other social insects (ants, bees, etc.), and that the architecture of the chemoreceptor system plays an important role in the formation of polyethism and community building.

## CONCLUSION

Insects possess a complexly organized chemoreception system based on proteins that belong to three superfamilies (*Table 4*). A characteristic feature of this system is lack of a strict correspondence between the receptor type and its functional role. Thus, ionotropic receptors (IRs) are involved both in the sense of smell (acid odors, amine odors) and taste perception (low concentrations of sodium chloride). The situation is similar with GR receptors, which, as their name suggests, are mainly involved in taste perception (bitter and sweet taste), while at the same time they can also be involved in olfaction (carbon dioxide). Only odorant receptors (ORs) are strictly olfactory.

**Table 4:** Comparative characteristic of the three main groups of insect chemoreceptors

Chemoreceptor superfamily	ORs (odorant receptors)	IRs (ionotropic receptors)	GRs (gustatory receptors)
Function in insect chemoreceptor system	Food odor and pheromone perception	Odor perception (acids and amines) and low-salt taste perception	Taste perception and carbon dioxide sensing
Protein quaternary structure/oligomeric status	heterodimers	• heterotetramers • heterodimers (acidic odors)	• monomers (Gr43a-like receptors) • heterodimers (sweet taste, carbon dioxide)
Response mechanism	ionotropic + metabotropic	ionotropic	• ionotropic (Gr43a-like receptors) • metabotropic (carbon dioxide sensing receptors)
Type of sensory neurons responsible for signal transduction in the central nervous system	Olfactory sensory neurons – OSNs		Gustatory receptor neurons – GRNs
Localization of sensory neurons in insects	Appendages of the forehead, antennae, maxillary palps		Legs and wings
Model systems used for the conducted studies	• Drosophila melanogaster “empty neuron”* • Xenopus laevis oocyte • Mammalian cells (HEK293)	• Drosophila melanogaster “empty neuron”* • Xenopus laevis oocyte	• Drosophila melanogaster “empty neuron”* • Xenopus laevis oocyte • Mammalian cells (HEK293)

^*^Drosophila melanogaster olfactory neuron lacking an endogenous receptor.

The architecture of the chemosensory system reflects the development of the evolutionary adaptations that allow insects to accurately and adequately respond to external chemical stimulation. Thus, GR and IR receptors demonstrate complementary sensitivity to carbon dioxide and acidic odors: low carbon dioxide concentrations are recognized by GR heterodimers (e.g., Gr21a and Gr63a in *Drosophila*), whereas high CO_2_ concentrations are recognized by IR heterodimers (IR8a-IR64a in *Drosophila*). The olfactory receptors of the IR and OR families, in turn, demonstrate complementary specificity and unequal sensitization ability, which apparently enables insects to accurately determine changes in the concentration of specific odorants even in the presence of a wide range of “background” molecules.

Evolutionary adaptations would probably also include unusual signal transduction mechanisms characteristic of insect chemoreceptors. For instance, odorant receptors (ORs) use both the ionotropic and metabotropic pathways of “chemical” signal transduction. The first way is important probably for a quick response to high concentrations of odorant, while the second one provides signal amplification when recognizing weak odors. The molecular mechanisms of IRs and GRs functioning have been studied much less, but the available data generally indicate a preferentially ionotropic transduction pathway of their signal. This, however, does not exclude the presence of alternative mechanisms. Thus, carbon dioxide receptors from the GR superfamily are characterized by a metabotropic response mediated by Gαq proteins and activating ion channels of the TRP family. It has been suggested that olfactory IR receptors can also interact with G proteins [55].

An ionotropic signal transduction pathway is quite common among all types of insect chemoreceptors. This fact is responsible for the significant peculiarity of their chemosensory system. However, we would like to note that a significant amount of blank spots remains on the “chemoreceptor map” of arthropods in general and insects, in particular. Both the specificity, molecular structure, and the signaling pathways of these receptors are still being studied.

## Abbreviations

OSN: olfactory sensory neuron
OR: odorant (olfactory) receptor
GPCR: G protein-coupled receptor
DAG: diacylglycerol
IP3: inositol trisphosphate
IR: ionotropic receptor
ATD: amino terminal domain
GRN: gustatory receptor neuron
GR: gustatory receptor
